# IR-NMR multimodal computational spectra dataset for 177K patent-extracted organic molecules

**DOI:** 10.1038/s41597-025-05729-8

**Published:** 2025-08-07

**Authors:** Federico Zipoli, Marvin Alberts, Teodoro Laino

**Affiliations:** 1 IBM Research Europe, Saümerstrasse 4, 8803 Rüschlikon, Switzerland; 2 NCCR Catalysis, Zurich, Switzerland; 3 University of Zurich, Department of Chemistry, Winterthurerstrasse 190, 8057 Zurich, Switzerland

**Keywords:** Infrared spectroscopy, Solution-state NMR, Computational chemistry, Density functional theory, Molecular dynamics

## Abstract

The construction of predictive models in molecular science increasingly relies on large, high-quality datasets. Synthetic data generation is becoming a foundational strategy for advancing model accuracy and enabling fast discovery workflows. To support the development of structure elucidation and spectral property prediction models, we present a comprehensive synthetic dataset of infrared (IR) and nuclear magnetic resonance (NMR) spectra for a diverse ensemble of organic molecules. The data were generated using a hybrid computational approach that integrates molecular dynamics (MD) simulations, density functional theory (DFT) calculations, and machine learning (ML) models. The dataset primarily consists of IR spectra for 177,461 molecules, derived from long-timescale MD simulations with ML-accelerated dipole moment predictions. In addition, it includes a smaller subset of ^1^H-NMR and ^13^C-NMR chemical shifts for 1,255 molecules. This unique combination of spectral data offers a valuable resource for benchmarking and validating computational methodologies, developing and enhancing artificial intelligence (AI) models for molecular property prediction, and facilitating the interpretation of experimental spectroscopic results. The dataset is publicly available through Zenodo, encouraging its broad utilization within the scientific community.

## Background & Summary

Molecular spectroscopy plays a vital role in probing chemical structure and dynamics, making it indispensable in both experimental and computational chemistry. Techniques such as infrared (IR) and nuclear magnetic resonance (NMR) spectroscopy are widely employed to investigate functional groups, bonding environments, and conformational features.

A central challenge is the accurate prediction of spectra from molecular structure. Equally important is the reconstruction of molecular structure from observed spectra. These two complementary directions form the foundation of data-driven spectral analysis frameworks^1^.

Recent developments in high-throughput spectroscopy and deep learning have enabled large-scale modeling of these correlations, offering significant improvements in prediction accuracy and opening new avenues for applications such as spectral database search, compound identification, and de novo molecular design.

The availability of large-scale experimental spectroscopic datasets has been instrumental in advancing these computational approaches. For infrared spectroscopy, the NIST Chemistry WebBook provides IR spectra for over 16,000 compounds^2^ many of these are sourced from the Coblentz Society collection^3^, while the NIST Quantitative Infrared Database^4^ focuses on high-resolution absorption coefficient spectra of volatile organic compounds. Other databases such as the Japanese AIST Spectral Database for Organic Compounds (SDBS)^5^ and Wiley’s commercial KnowItAll spectral collections^6^ have further expanded the available experimental IR data. In the NMR domain, publicly accessible resources include specialized datasets like nmrshiftdb2 for small organic molecules^7^, the ACS Spectroscopy Data for Teaching collection^8^, and more recently, the 2DNMRGym dataset^9,10^ which provides over 22,000 annotated HSQC spectra specifically designed for machine learning applications in 2D NMR analysis. While these experimental repositories serve as crucial benchmarks for validating computational predictions and training data-driven models, their limited scope and licensing constraints have motivated the development of synthetic spectral data generation methods.

Complementing these experimental resources, accurate simulation of molecular spectra not only underpins compound identification, structure elucidation, and materials discovery, but also enables large-scale synthetic data generation, which is crucial for bypassing the twin bottlenecks of slow, labor-intensive spectral acquisition and the scarcity of experimental datasets with friendly-licensing terms for AI applications. First-principles methods such as density functional theory (DFT) offer high-quality predictions, but their high computational cost limits their scalability to large datasets or high-throughput workflows. Moreover, most computed IR spectra are obtained within the harmonic approximation, in which the Potential Energy Surface is truncated at second order (Hessian matrix). Because this neglects higher-order (anharmonic) terms, it fails to capture overtone/combination bands and anharmonic mode coupling, leading to systematic frequency shifts and intensity errors when compared to experimental results that are particularly noticeable in the crowded fingerprint region.

To address these challenges, we present a dataset that uniquely consists of anharmonic IR spectra, derived from molecular dynamics (MD) simulations coupled with machine learning (ML)-accelerated dipole moment predictions. For a subset of molecules we also provide DFT-based nuclear magnetic resonance (NMR) chemical shifts. This approach for IR spectra captures coupled vibrational modes and anharmonic behavior arising from thermal sampling and mode coupling, natively and without relying on finite-difference approximations or explicit Hessian evaluation. In parallel, for NMR, we provide proton (^1^H) and carbon (^13^C) chemical shifts computed from DFT on conformations sampled along MD trajectories, thereby introducing realistic thermal effects into the chemical shift predictions. This ensemble-based method captures structural diversity beyond single optimized geometries and supports more transferable learning models.

By integrating anharmonic IR and DFT-based NMR data within a unified, high-quality dataset, we aim to support a broad range of use cases including benchmarking of computational methodologies, AI model development, and experimental spectrum interpretation. Several purely computational vibrational-spectroscopy resources have already appeared in Scientific Data: the 5,099 compound Raman database for inorganic crystals by Bagheri & Komsa^11^; Li *et al*.’s hybrid-functional Raman set for 161 inorganic solids^12^; the 3,504 entry library for two-dimensional materials ^13^; and the earlier 55-compound benchmark of Liang *et al*.^14^. All of these datasets are limited to harmonic approximation simulations and lack any NMR information. Very recently, Liang *et al*.^15^ released the IR-Raman ChEMBL dataset, which, on par with the approximations adopted in the previous datasets, provides harmonic IR and Raman spectra for approximately 220,000 drug-like molecules extracted from ChEMBL. In contrast, our USPTO-Spectra dataset^16^ supplies anharmonic IR spectra obtained from ab initio molecular-dynamics trajectories together with DFT-level ^1^H/^13^C NMR shifts for 1,255 patent-derived molecules, offering an higher quality multimodal benchmark tailored for multimodal IR-NMR machine-learning tasks^17^. The combined availability of IR and NMR spectra in this dataset therefore opens new possibilities for training, benchmarking, and validating multimodal and multitask foundation models, to jointly interpret vibrational and magnetic resonance signals, leading to more robust spectral prediction and structure elucidation. The dataset is openly available via Zenodo^16^ and we hope it will catalyze the further development of interpretable, data-driven tools in spectroscopy-driven molecular discovery.

## Methods

### Molecular Selection

We started from a pool of approximately 200,000 molecules sampled from the USPTO dataset^18^. Drawn from patent literature, the USPTO dataset^18^ features molecules that closely mirror those prevalent in industry and, to a lesser extent, academia^19^. From this initial set, we filtered by retaining only those composed of the following elements: B, Br, C, Cl, F, H, I, N, O, P, S, and Si and within a heavy atom count (all atoms except for hydrogen) of 5–35. The SMILES representations were then converted into XYZ coordinates using RDKit^20^. We encountered cases where some molecules couldn’t be processed successfully by RDKit or by subsequent scripts designed to generate GAFF input for LAMMPS due to various computational limitations and time constraints. In addition, molecules containing Si and B-atoms could not be successfully parameterized using GAFF2 via the Antechamber toolchain, further reducing the effective number of molecules available for simulation down to 177,461 molecules.

### Computational Approach for IR Calculation

Finite-difference approaches inherently assume a harmonic potential and thus fail to capture anharmonic contributions to the IR spectrum, such as mode coupling. To overcome this limitation, we computed the IR spectrum from the dipole-dipole autocorrelation function obtained from molecular dynamics (MD) simulations at room temperature, which intrinsically accounts for these anharmonic effects.

First-principles simulations based on density functional theory (DFT) provide a reliable framework for generating both accurate MD trajectories and computing dipole moments. However, their high computational cost makes them impractical for large-scale or long-time MD simulations, which are essential for high-throughput or statistically converged spectral analysis.

To address this limitation, we adopted a hybrid approach. We used classical MD to generate long trajectories efficiently, and evaluated dipole moments using DFT on a subset of snapshots sampled at regular time intervals. To further accelerate the process, we trained a machine learning (ML) model on the DFT-computed dipole data generated in this work. This model is specifically designed to accelerate IR spectra generation within a defined chemical space and is not intended for out-of-domain generalization to entirely novel scaffolds. This model enables fast and accurate dipole predictions across the full trajectory, significantly boosting throughput without compromising on accuracy, see the technical validation section.

#### Classical MD

We employed the second-generation Generalized Amber Force Field (GAFF2)^21^ with the LAMMPS code^22^ to perform classical molecular dynamics (MD) simulations of isolated molecules in vacuo. For each molecule in our dataset^16^, we generated a GAFF2-compatible input using an automated input-generation pipeline. Each molecule underwent MD simulations under the following conditions: the system was first equilibrated in vacuo at 300 K using a Langevin thermostat with a damping constant of 0.1 ps for 25 ps, using a time step of 0.5 fs. This was followed by a 100 ps production run in the microcanonical (NVE) ensemble, also in vacuo. During the production run, classical trajectories were recorded every 2.5 fs to resolve high-frequency vibrational modes, and were subsequently used to compute the dipole-dipole autocorrelation function required for IR spectrum generation. In parallel, classical dipole moments were sampled on-the-fly every 1 fs to enable direct spectral reconstruction.

As discussed in the following sections, we used DFT to compute reference dipole moments on selected snapshots and trained a machine learning model to predict dipoles across the full trajectory, thereby enhancing spectral accuracy while maintaining computational efficiency.

#### DFT Calculations and Wannier Analysis

To obtain accurate dipole moments, we performed first-principles calculations on snapshots extracted from the classical MD trajectories using density functional theory (DFT) as implemented in the CPMD code^23,24^. We employed the PBE exchange-correlation functional^25^ together with Goedecker-Teter-Hutter (GTH) pseudopotentials^26^, using a plane-wave cutoff of 100 Ry for the wavefunction and 400 Ry for the electron density.

On these selected snapshots, we further performed Wannier function analysis to compute both the total and local dipole moments of the molecules. This analysis provides insight into charge localization and polarization effects via the maximally localized Wannier centers^27^. These DFT-based dipole moments served as reference data for training our machine learning model.

In parallel, and for benchmarking purposes, we also performed first-principles Born-Oppenheimer (BO) molecular dynamics simulations on a selected subset of molecules using the same DFT settings described above. These BO-MD trajectories served as a reference to assess the quality of both the classical MD and the machine learning-based dipole predictions.

#### ML for dipole prediction

We employed the Deep Potential (DP) framework as implemented in DeePMD-kit to construct a deep neural network potential^28–31^. The model was trained on atomic configurations containing the elements B, Br, C, Cl, F, H, I, N, O, P, S, and Si. The descriptor used was the Deep Potential Smooth Edition (DeepPot-SE)^32^ environment-dependent descriptor (type se_e2_a), with a species-dependent neighbor count (sel) of up to 84 atoms within a cutoff radius (rcut) of 6.0 Å and a smoothing cutoff (rcut_smth) of 1.0 Å. Further details, including the full DeepMD-kit input script, are provided in the Supporting Information. We determined the maximum number of neighbors to include in the sel count by computing the maximum number of neighbors in our configurations. For each species, we used a specific count augmented by 2–3 atoms to provide a margin. This resulted in the following values: 66, 60, 84, 68, 62, 84, 66, 78, 84, 84, 68, and 64 for B, Br, C, Cl, F, H, I, N, O, P, S, and Si, respectively. While Si and B atoms were excluded from the GAFF-based dataset, we retained their placeholder entries in the DeepMD-kit input files to allow for potential fine-tuning of the model with these additional species.

The descriptor network consisted of three layers with 32, 64, and 128 neurons, respectively, using a ResNet architecture with resnet_dt enabled. The axis embedding dimension was set to 16 neurons, and the model was trained in 32-bit floating point precision (float32). Neighbor type selection was restricted to one side (type_one_side: true).

The fitting network was specifically configured to predict dipole moments (type: dipole) using all defined atomic species. It also followed a ResNet architecture with three hidden layers of 128 neurons each, and used the same floating-point precision and random seed (seed: 1) as the descriptor.

The model was trained using a learning rate that decays exponentially (type: exp), starting from 1.0 × 10^−2^ ending at 1.0 × 10^−8^ with a decay step size of 2,000,000. The loss function was tensor-based and used equal weights (pref = 1.0, pref_atomic = 1.0) for total and atomic contributions.

**Fig. 1:**
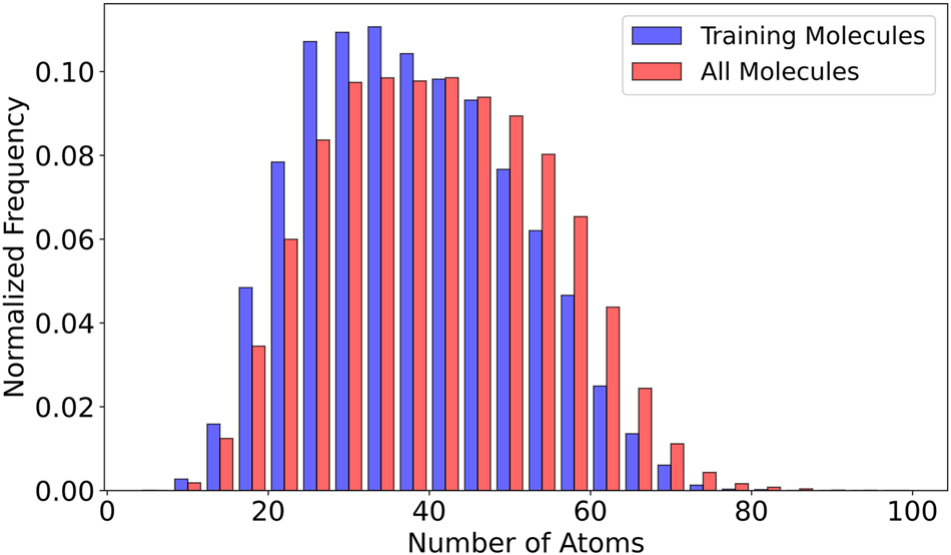
Histogram comparing atom counts between the 15,311 training molecules and the complete set of 177,461 molecules. The blue bars represent the frequency distribution for the training set, while the red bars correspond to the all-molecule dataset (including train set). The plot shows the overall distribution of atomic sizes across both sets.

We incrementally fine-tuned the model by adding new training configurations as they became available from ongoing DFT computations. For the fine-tuning, we set an initial learning rate equal to 1.0 × 10^−4^. In total, training was conducted for 6,000,000 steps. Training data was fed in batches of 20 configurations, while validation was performed every few steps using 3 batches of 3 configurations each. The batch size was limited to 20 because, in DeePMD-kit, batches are not sampled across different molecules. In the last fine-tuning we used the largest training dataset consisting of 15,311 molecules, using 20 configuration per molecule. Figure 1 illustrates the distribution of the number of atoms for those molecules.

Model checkpoints were saved every 10,000 steps. We monitored training progress using a validation set larger than performed directly within the DeePMD-kit input. This set consisted of about 2,000 frames taken from both previously seen molecules (at different snapshots along the trajectories) and from unseen ones. We logged the training progress every 100 steps, we used a fixed random seed (seed: 10) ensured reproducibility across training runs.

##### Train set selection

We used the Tanimoto similarity applied to Morgan fingerprints to select a representative group of molecules to serve as the training set. The dipole moment of each molecule in this set was then computed using DFT.

To ensure good coverage of chemical space, we progressively expanded the training set. To maximize coverage while minimizing the number of molecules, we applied k-means clustering to the Morgan fingerprints, choosing 10,000 clusters and ensuring that at least one molecule was selected from each cluster. As we expanded the training dataset, molecules from these additional regions of chemical space were included in subsequent model fine-tuning steps. In the final refinement 15,311 molecules were included in the training set.

**Fig. 2:**
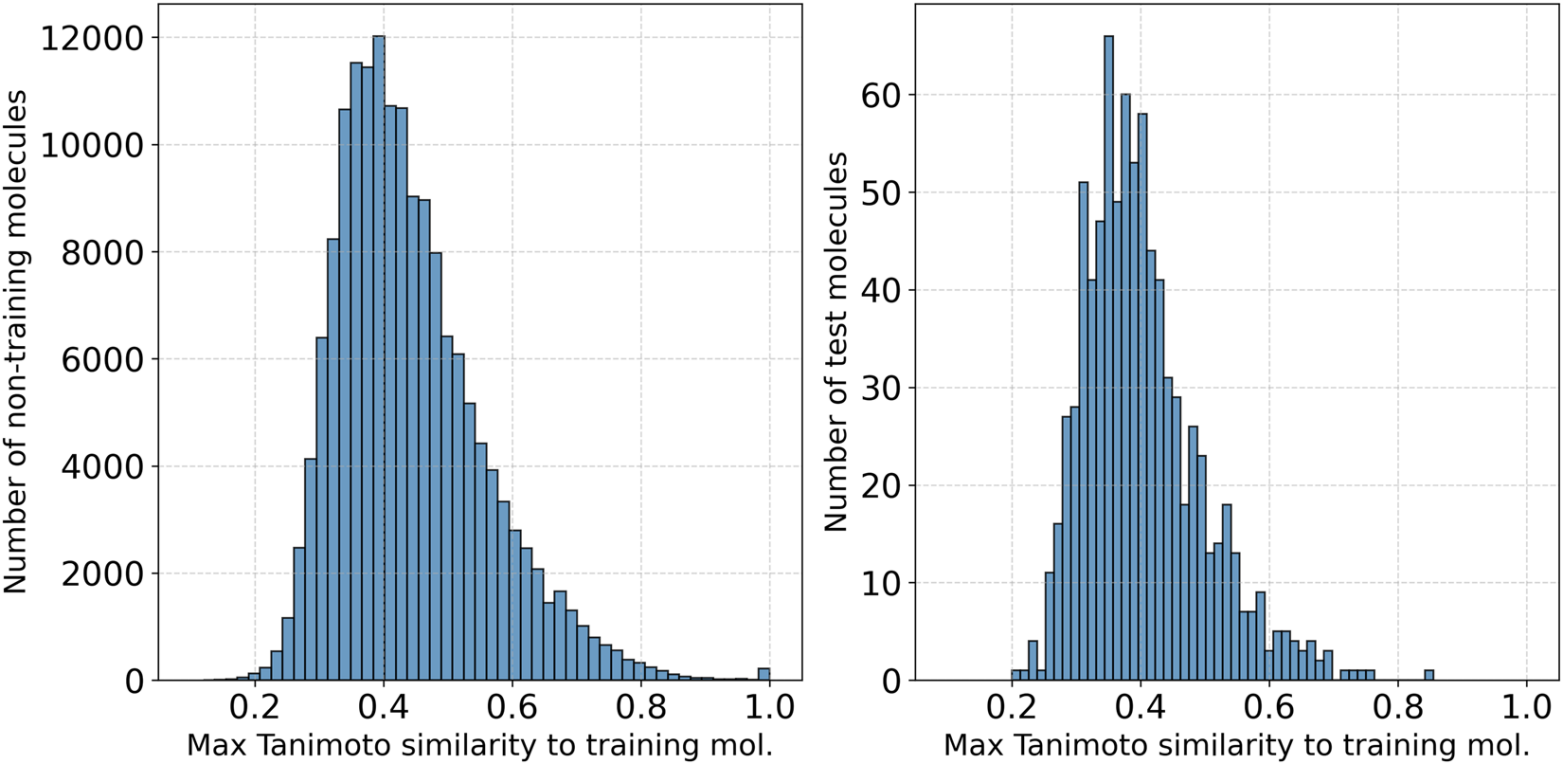
(Left) Distribution of maximum Tanimoto similarity between molecules not used for training (162,150 molecules) and those in the training set (15,311 molecules). (Right) Distribution of maximum Tanimoto similarity between molecules in the test set (841 molecules) and those in the training set (15,311 molecules). The comparable distributions demonstrate that our randomly selected test set provides a representative sample for evaluating model performance on unseen molecules within the same chemical space.

Figure 2 shows the distribution of Tanimoto similarity scores between molecules not used for training and those in the training set. The left panel displays the distribution for all unused molecules, while the right panel shows the distribution specifically for the test set. For each molecule, we computed the maximum similarity to any molecule in the training set. A higher similarity score indicates closer molecular resemblance. These comparisons are made based on molecular fingerprints rather than scaffold structures.

For the complete set of unused molecules, we find that 57.4 % have a Tanimoto similarity greater than 0.4, and 24.5 % exceed a similarity of 0.5 relative to the training set. Similarly, for the test set molecules, 44.0 % have a Tanimoto similarity greater than 0.4, and 13.7 % exceed a similarity of 0.5 relative to the training set. The comparable distributions between the unused molecules and test set confirm that the test set is representative of the broader chemical space not used for training.

**Fig. 3:**
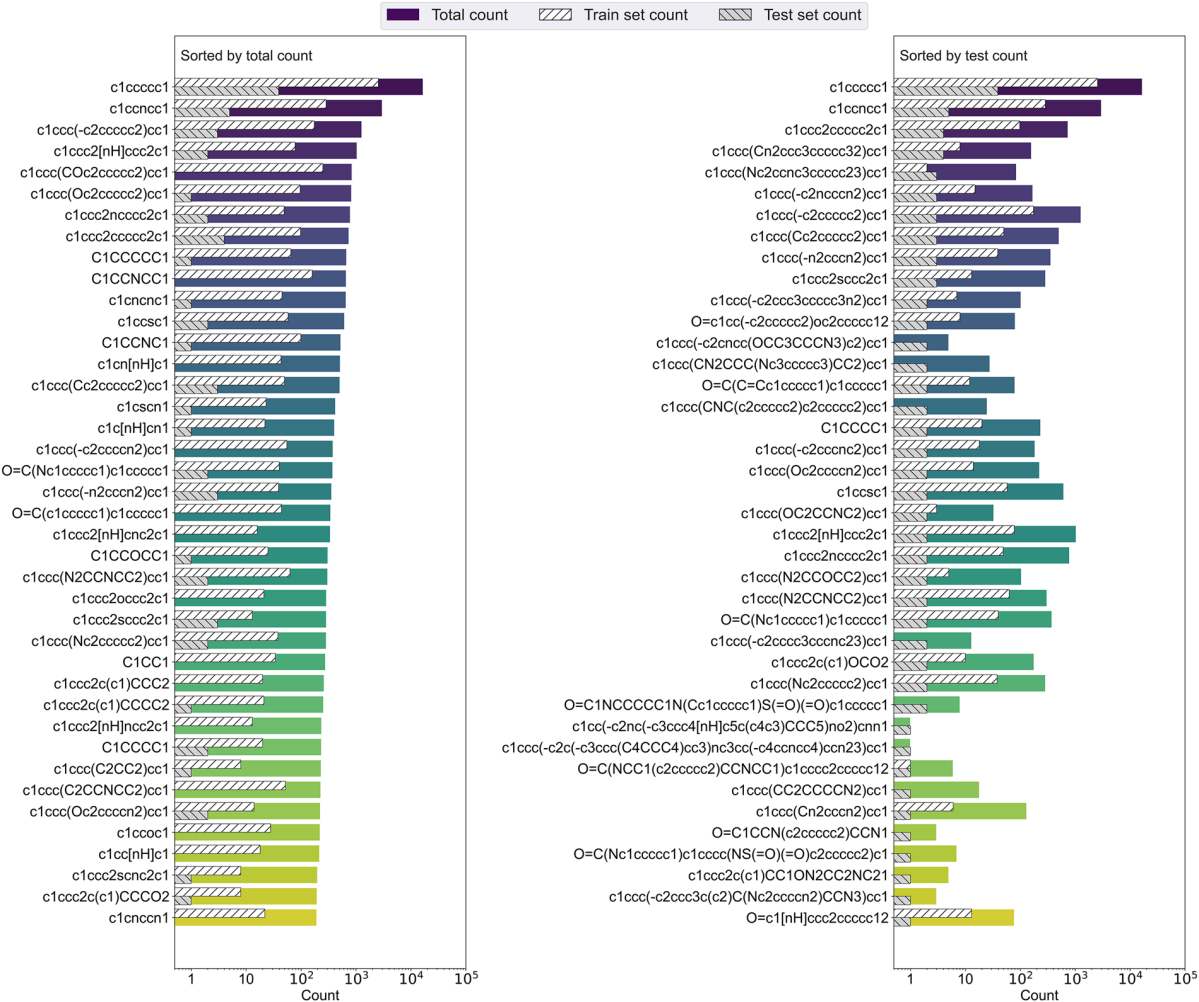
Left barplot shows the top 40 most frequent molecular scaffolds selected from over 60,000 unique structures. Right barplot shows the same data but ranks scaffolds by their frequency in the test set. Bar segments with hatching indicate how many examples are present in the training (white background) and test (gray background) sets, revealing the coverage of top scaffolds in model development.

A UMAP projection of Morgan fingerprints for the scaffold molecules, along with a visualization of the subset used for dipole training, is provided in Figure S1 (Supporting Information) to illustrate coverage in the embedding space. To better understand the structural diversity of our dataset^16^, we analyzed the distribution of molecular scaffolds and identified the 40 most frequently occurring ones out of more than 60,000 unique entries. Figure 3 shows two complementary views of scaffold distribution. The left panel highlights the top 40 scaffolds by their total frequency across the dataset, while the right panel shows the same scaffolds ranked by their frequency in the test set. Each bar is split to show the number of instances present in the training and test sets (hatched segments) versus the total count, allowing us to assess how well the training data captures common structural motifs. The scaffold analysis reveals important characteristics of our train-test split. Among the 746 unique scaffolds in the test set, only 155 are also present in the training set, meaning 591 scaffolds (about 80%) in the test set are completely absent from the training data. This scaffold diversity in the test set, combined with the comparable Tanimoto similarity distributions shown in Fig. 2, demonstrates that our random molecule selection produces a representative test set that includes both familiar molecular frameworks and novel scaffolds not encountered during training.

##### High throughput computation of IR spectra

We computed IR spectra for 177,461 molecules using dipole moments predicted via DeePMD-kit along classical MD trajectories. Although the model significantly accelerates predictions compared to ab initio methods, generating dipole moments for every time step remains comparatively expensive. To ensure sufficient spectral resolution while maintaining computational efficiency, we sampled the MD trajectories every 2.5 fs for all molecules. This sampling interval enables accurate reconstruction of vibrational modes up to  ≈6600 cm^−1^, well above the 3600–3700 cm^−1^ upper limit typically observed for IR-active vibrations in vacuum. The chosen resolution thus ensures that high-frequency features, such as O-H or N-H stretches, are properly captured across the dataset.

### Computational Approach for NMR Calculation

To complement the IR dataset, we also generated a Nuclear Magnetic Resonance (NMR) dataset using the same set of molecular systems and computational framework. The NMR calculations were performed on molecular snapshots taken from the same MD trajectories used in the IR study. While we initially aimed at a larger set of molecules (approx. 5,000-10,000), the significantly higher computational cost of NMR calculations—particularly the need to average over ten conformational frames per molecule—necessitated stopping data collection once a sufficient number of jobs converged. Our final NMR dataset comprises 1,255 unique molecules after canonicalization.

All NMR calculations were performed using DFT with the same computational setup described in the IR section. In addition to sampling dynamic snapshots from molecular dynamics trajectories, we also carried out geometry optimizations to obtain relaxed structures for each molecule. These optimized geometries served as references for static NMR calculations and offered insight into the influence of thermal motion on predicted chemical shifts by comparing room-temperature ensemble averages to zero-temperature equilibrium structures. Moreover, relaxed geometries facilitated the identification of symmetry-equivalent atoms, which was essential for averaging of chemical shifts.

The MD snapshots were generated from classical MD simulations under the same setup as the IR calculations, typically spanning 100 ps with frames sampled every 5 ps. For each molecule, we aimed to compute NMR properties on about 20 snapshots; however, to complete the calculations in a reasonable time, we typically used the first 10 converged snapshots. These usually correspond to the initial frames of the trajectory, but some variability exists due to occasional convergence issues or computational constraints. All simulations correspond to isolated molecules at approximately 300 K, providing a realistic dynamic ensemble for NMR prediction.

The dataset includes both the relaxed atomic positions and the coordinates for each MD frame. Additionally, we provide the raw CPMD NMR output files, including the TMS-referenced chemical shifts. An example is provided in the Supporting Information.

#### Post-Processing of NMR Calculations

To facilitate comparison with experimental data and support efficient machine learning tasks, the raw shielding tensors were converted into averaged chemical shifts. Equivalent hydrogen atoms—either bonded to the same carbon (including methyl groups where hydrogens are chemically equivalent) or related by molecular symmetry—were identified and grouped, and their chemical shifts were averaged accordingly. A similar grouping and averaging procedure was applied to symmetry-equivalent carbon atoms. This averaging was performed at two hierarchical levels: first, across molecular dynamics frames to capture thermal fluctuations and conformational diversity; and second, across symmetry-equivalent atoms within each frame to respect molecular symmetry and chemical equivalence.

In addition to mean chemical shifts, we compute the standard deviation of shifts at both levels to capture variability due to dynamic effects and subtle differences among equivalent atoms. This statistical treatment enables a more comprehensive characterization of NMR parameters, supporting benchmarking against experiments and robust model training that incorporates uncertainty and molecular flexibility. Detailed statistics, including maximum deviations and illustrative figures, are presented in the Data Records section.

#### Reference for NMR Calculations

All chemical shifts were referenced against tetramethylsilane (TMS), a widely used internal standard in NMR spectroscopy. The chemical shifts reported for hydrogen and carbon atoms were obtained by subtracting the DFT-predicted shielding values of TMS from those of each atom, consistent with the experimental conventional practice.

## Data Records

The dataset is hosted on Zenodo and available for download at 10.5281/zenodo.15669241^16^. The dataset^18^ includes infrared (IR) spectral data derived from molecular dynamics simulations combined with dipole moment analyses, as well as nuclear magnetic resonance (NMR) spectra computed from a subset of the same MD trajectories.

### IR Dataset

The IR spectra of 177,461 molecules are stored as compressed pandas DataFrames in Apache Parquet format. Due to practical limitations when loading the entire dataset from a single file, we divided the dataset into nine chunks: eight files each containing 20,000 records, and a final file with the remaining molecules. This format ensures efficient access and compatibility with common data analysis workflows.

Each entry in the dataset corresponds to a distinct molecule. A description of the dataset structure is provided in Table 1. IR spectra are computed from the dipole moment time series following the methodology described by Braun^33^. Dipole moments were sampled every 2.5 fs over 100 ps, resulting in 40,001 time steps per trajectory. To reduce edge effects in the Fourier transform, a Blackman window was applied to the second half of the autocorrelation function, as proposed in Ref. ^34^ using the following equation

1 $$w[n]=0.42\,-\,0.5\,\cos (\frac{2\pi n}{N-1})+0.08\,\cos (\frac{4\pi n}{N-1}),\quad 0\le n\le N\,-\,1,$$

where *N* is the total number of autocorrelation points. To ensure comparability with experimental IR spectra, we applied quantum correction factors following the approach of Gaigeot and Sprik^35^ at 300 K. The resulting spectra have a frequency resolution of approximately 0.3336 cm^−1^. For storage efficiency, only the first 12,000 frequency points are retained, covering up to 4003.1694 cm^−1^. This range comfortably includes all physically relevant vibrational modes in vacuum, which typically do not exceed 3700 cm^−1^.

**Table 1:** Description of the data fields in the IR spectra dataset.

Column	Description
id	Unique molecule identifier
smiles	SMILES representation of the molecule
type	Atom types per molecule (list of integers)
Frequency (cm^−1^)	IR frequency axis in wavenumbers
ir_spectra	IR spectrum (quantum corrected)

The atomic type integers in type correspond to specific elements. The species-to-type mappings are B: 0, Br: 1, C: 2, Cl: 3, F: 4, H: 5, I: 6, N: 7, O: 8, P: 9, S: 10, Si: 11.

### NMR Dataset

The dataset provided in the nmr_dataset.parquet file contains 1,255 molecular structures along with computed NMR properties derived from molecular dynamics trajectories. Each row corresponds to a unique organic molecule identified by a distinct id, and includes metadata, structural data, and computed ^1^H and ^13^C chemical shifts sampled across multiple simulation frames. The key fields contained in the dataset are described in Table 2.

**Table 2:** Top-level and nested dictionary keys.

Key	Description
id	Molecule identifier
smiles	SMILES representation of the molecule
atoms	List of atomic symbols
xyz	Cartesian coordinates of atoms
c_atoms_group_index	Grouping of carbon atoms by symmetry
h_atoms_group_index	Grouping of hydrogen atoms by symmetry and CH_3_ attachment
frames	Dictionary of NMR results by frame index (e.g., ‘0’ to ‘9’)
↳ c_nmr_grouped_peaks	Grouped ^13^C NMR chemical shifts
↳c_nmr_peaks_ave	Averaged ^13^C NMR shifts per group
↳c_nmr_peaks_unsorted	Unsorted ^13^C NMR shifts listed per carbon atom, in the same order as the atoms in the xyz array
↳h_nmr_grouped_peaks	Grouped ^1^H NMR chemical shifts
↳h_nmr_peaks_ave	Averaged ^1^H NMR shifts per group
↳h_nmr_peaks_unsorted	Unsorted ^1^H NMR shifts listed per hydrogen atom, in the same order as the atoms in the xyz array
↳nmr_cpmd_text	NMR shielding CPMD output including TMS reference subtraction, see Table S1 in Supporting Information
↳xyz	Coordinates for this specific frame
number_of_frames	Number of sampled frames
frame_ids	List of frame identifiers
averaged_frames	Frame-averaged NMR shifts
h_nmr_std	Standard deviation of ^1^H shifts across frames
c_nmr_std	Standard deviation of ^13^C shifts across frames
h_nmr_max_std	Maximum standard deviation of ^1^H NMR in a group across frames
c_nmr_max_std	Maximum standard deviation of ^13^C NMR in a group across frames

This comprehensive dataset captures both structural and dynamic aspects of NMR parameters, enabling in-depth analyses of molecular behavior under realistic thermal conditions. It serves as a valuable resource for the development, validation, and benchmarking of machine learning models and computational methods in cheminformatics and NMR spectroscopy.

## Technical Validation

### Infrared (IR) Dataset

**Fig. 4:**
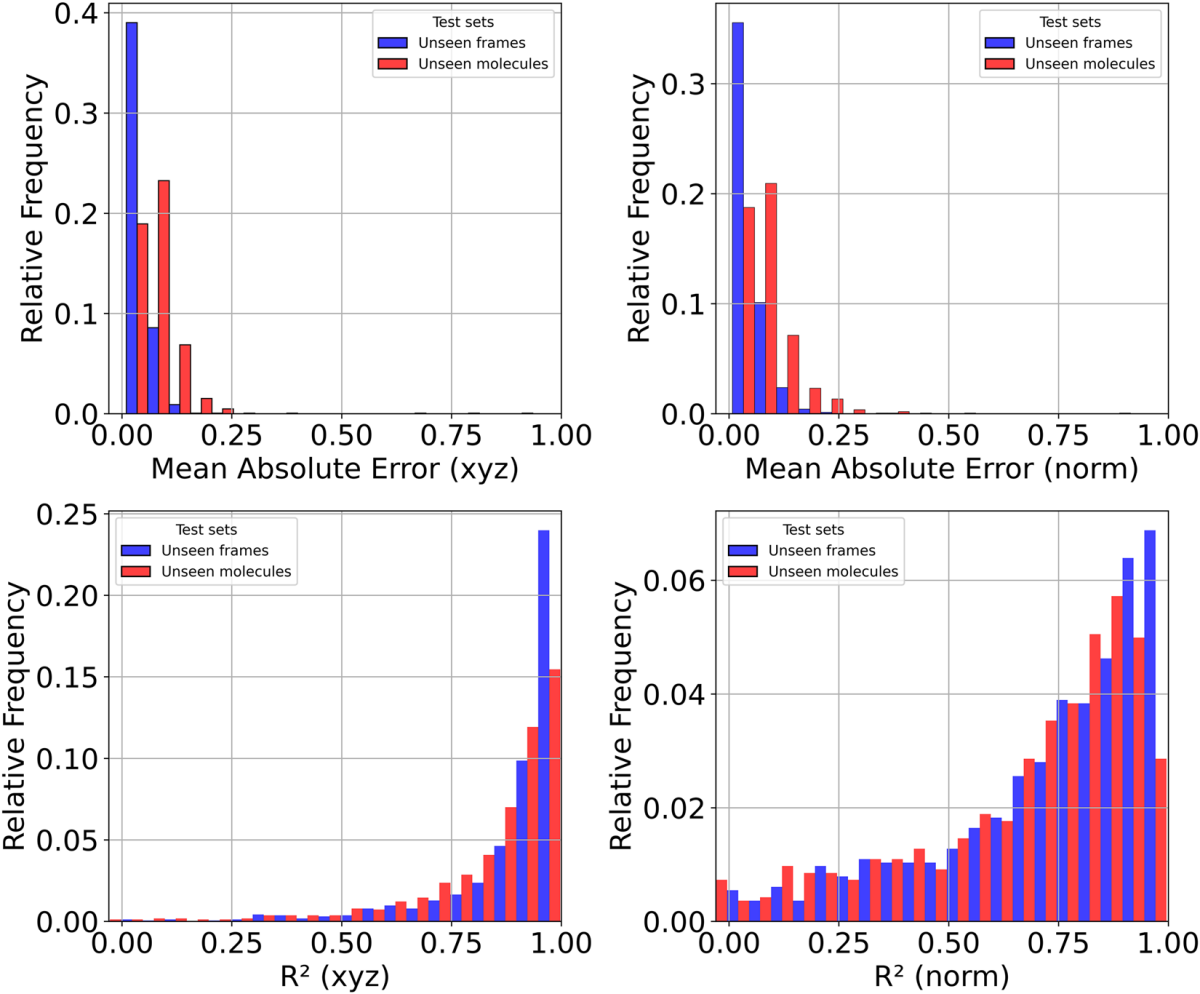
Distribution of Dipole Prediction Errors for Two Test Set Groups. Histograms show the distribution of prediction errors across four metrics: mean absolute error (MAE) for dipole vector components and norm, and R^2^ scores for both. Data are split between “Unseen frames” (previously seen molecules, new configurations) and “Unseen molecules” (entirely unseen molecules). Each subplot normalizes the count to show relative frequency, enabling a direct comparison of error distributions regardless of group size.

To assess the quality and reliability of our dipole moment predictions and the derived IR spectra, we performed a series of validation steps comparing the model’s outputs to first-principles reference data. We began by quantifying the mean absolute error (MAE) between dipole moments predicted by our machine learning model and those computed using DFT. This metric offers a direct, frame-wise measure of model accuracy and is reported across a diverse test set. As illustrated in Fig. 4, we divided the test data into two groups: “Unseen frames”, which consist of new configurations from molecular trajectories used in training (i.e., the molecules were seen during training), and “Unseen molecules”, which include entirely new molecules not encountered by the model during training.

**Fig. 5:**
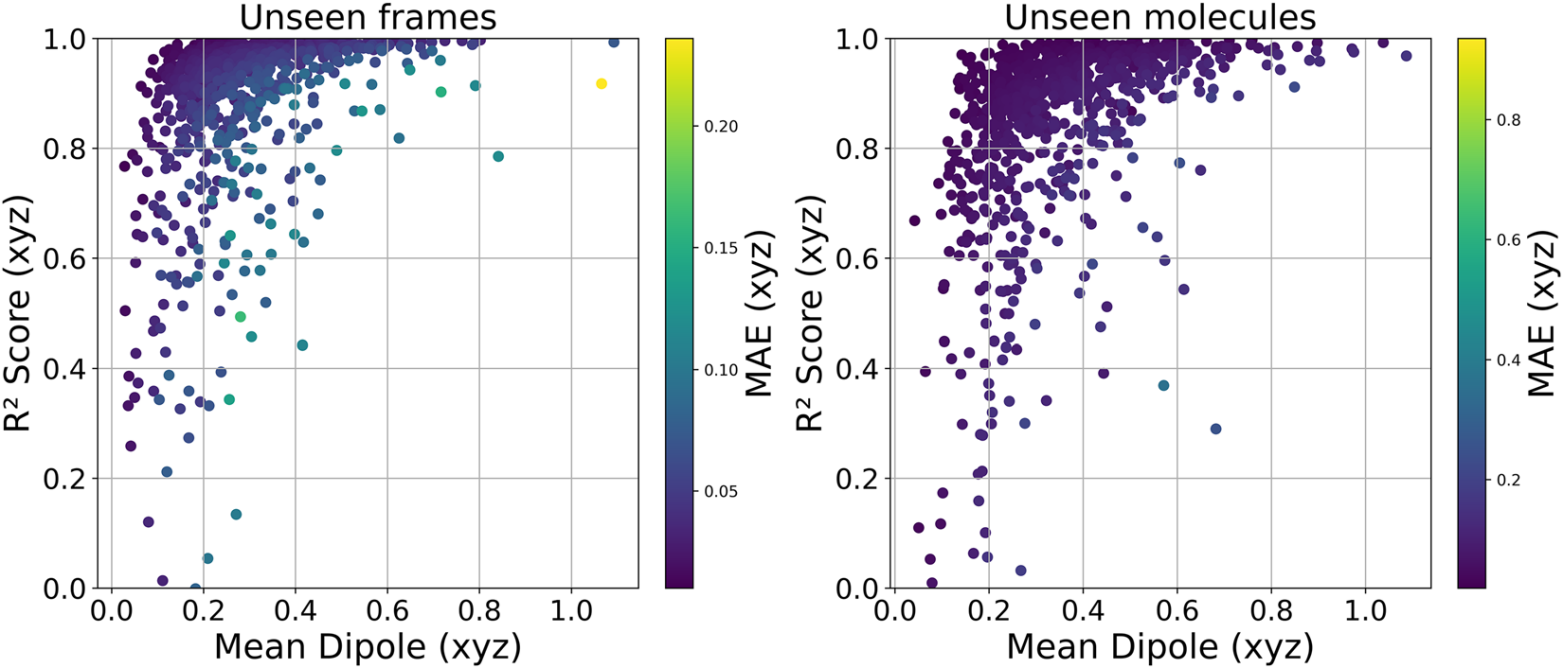
R^2^ score vs mean dipole magnitude for two test sets: unseen frames (left) and unseen molecules (right). The color of each point reflects the MAE (xyz). To improve interpretability, points are plotted in order of increasing MAE so that higher-error cases appear on top and are not hidden by overlapping more frequent lower-error points.

The R^2^ scores shown in Fig. 4 are, in some cases, close to zero. This behavior is observed for molecules with very low or nearly vanishing dipole moments, where even small absolute errors can result in poor R^2^ values. This is a known limitation of the R^2^ metric when the signal (i.e., the dipole moment) is weak or nearly absent. Figure 5 illustrates this correlation by plotting the R^2^ scores against the mean of the dipole moments for each molecule along the trajectory.

**Fig. 6:**
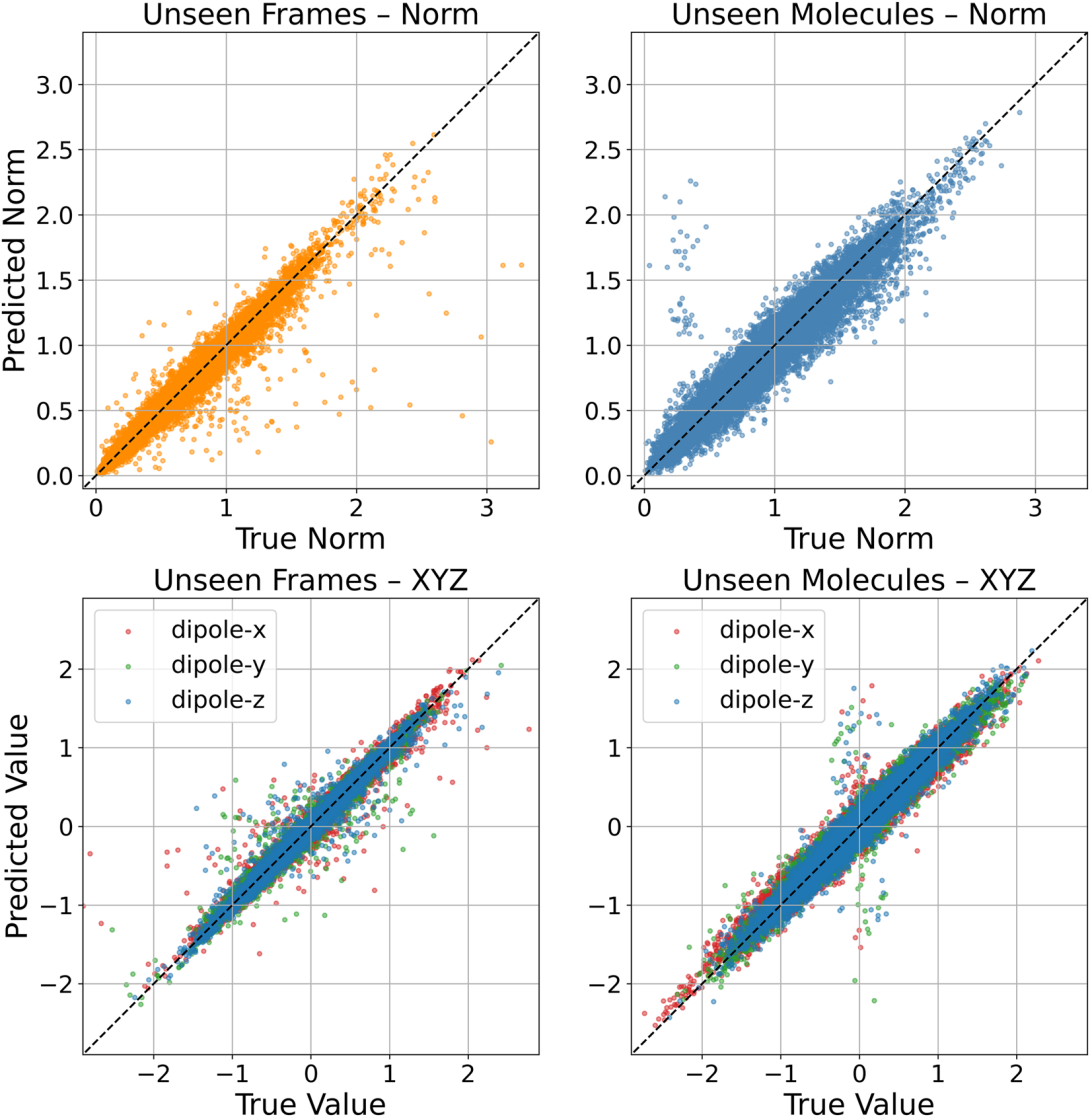
Comparison of predicted and ground truth dipole moments for unseen data. Top row: Scatter plots of dipole moment norms for individual frames (left) and whole molecules (right). The dashed black line indicates perfect agreement. Bottom row: Component-wise comparison of the *x*, *y*, and *z* components of the dipole moments for frames (left) and molecules (right), colored by axis. Deviations from the identity line reflect axis-specific prediction errors.

To complement the aggregate metrics shown earlier, Fig. 6 directly compares predicted and reference dipole moments for unseen data. The top row shows the agreement in dipole norm, while the bottom row breaks down the individual *x*, *y*, and *z* components. Overall, the model achieves strong agreement across both norms and components. However, in the component-wise plots for unseen molecules (bottom right), we observe a more pronounced deviation for two out of 843 test molecules: the predictions tend to yield small but non-zero values even when the true dipole components are close to zero.

**Fig. 7:**
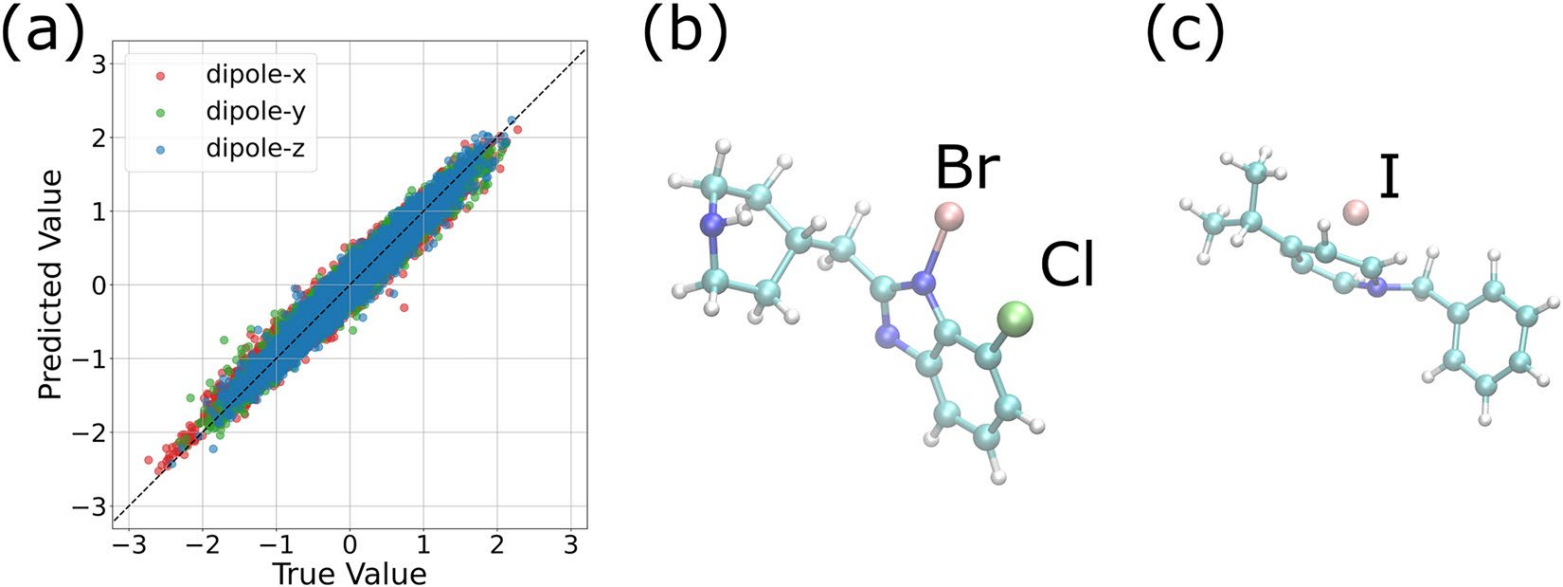
(**a**) Comparison of predicted and reference dipole moments for unseen data, excluding the two molecules with the largest deviations. (**b**) and (**c**) show the structures of these two outlier molecules: molecule ID 11726 in (**b**) and molecule ID 22447 in (**c**). Atom colors are as follows: carbon (C) in cyan, hydrogen (H) in white, nitrogen (N) in blue, chlorine (Cl) in light green, bromine (Br) in pink, and iodine (I) in pink. Halogen atoms are additionally labeled with their atomic symbols. The scatter plot in (**a**) presents a component-wise comparison of the *x*, *y*, and *z* dipole components, with each point representing a single frame. Deviations from the identity line highlight axis-specific prediction errors.

Figure 7 highlights these two molecules and re-displays the predicted versus true dipole moments after removing them. As shown, the agreement for the remaining 841 test molecules is consistently strong across all components. Upon inspection, both outlier molecules (panels b and c) contain unusual halogen substitutions, including bromine, chlorine, and iodine. These elements are underrepresented in the training set, and their presence likely introduces subtle distribution shifts. This suggests that the deviation arises not from classical overfitting, but from the model’s limited exposure to such chemistries. The small residual dipole values predicted by the model in otherwise symmetric cases may also point to limitations in handling near-zero signals or symmetry-related cancellations.

Together, these validation steps demonstrate that our model not only allowed the approximations of dipole moments with low error but also enables accurate computation of IR spectra across a diverse set of molecules and dynamic regimes. With this accuracy we generate more than of 177 thousands spectra.

**Fig. 8:**
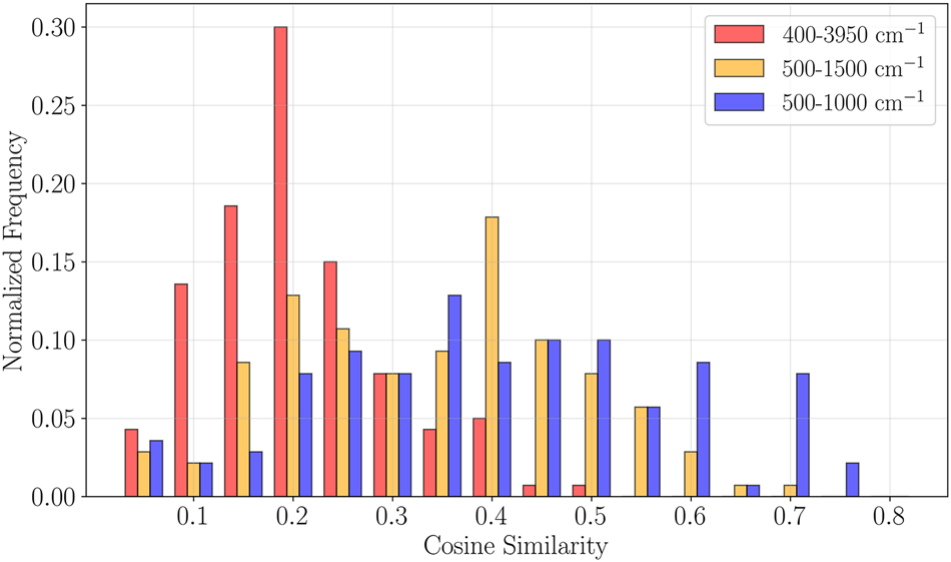
Normalised frequency of cosine similarity scores between experimental IR spectra of the 140 common molecules from the Spectroscopy Database^8^ and computed spectra from our dataset^16^. The analysis compares three frequency ranges: full spectrum (400–3950 cm^−1^, red bars, average similarity = 0.21), mid-infrared region (500–1500 cm^−1^, orange bars, average similarity = 0.34), and fingerprint region (500–1000 cm^−1^, blue bars, average similarity = 0.40). The improved agreement at lower frequencies reflects the reduced influence of hydrogen-bond interactions, which complicate experimental comparisons at higher frequencies. All distributions are normalized to show relative frequency of occurrence.

Beyond the overall accuracy, we also conducted an error analysis for our IR data. Figure 8 illustrates this analysis, which was performed on 140 molecules that overlap between our dataset^16^ and the Spectroscopy Database^8^. Our analysis reveals an average cosine similarity of 0.21 across the full 400–3950 cm^−1^ range. This similarity improves to 0.34 when focusing on the 500–1500 cm^−1^ range, and further to 0.40 for the 500–1000 cm^−1^ range. This improvement is observed because higher frequencies are predominantly affected by hydrogen-bond interactions, which complicate experimental comparisons.

It is important to acknowledge certain challenges and limitations in this analysis. First, this evaluation was performed on a relatively small subset of only 140 simple molecules, compared to the 177,461 molecules in our full dataset. This limitation stems from the scarcity of experimental IR data available for comparison, often due to restrictive licensing. Second, and crucially, our computational IR spectra are obtained in vacuo, whereas the experimental spectra in the Spectroscopy Database were acquired using Fourier transform infrared attenuated total reflectance of liquids/solids. This fundamental difference means that the experimental spectra are significantly influenced by hydrogen bonding and other condensed-phase effects, particularly at higher frequencies. As evidenced by the increasing similarity observed in Fig. 8 when narrowing the spectral range towards the fingerprint region (e.g., 500–800 cm^−1^), the impact of these environmental factors on higher frequencies is substantial, highlighting the need for careful consideration in experimental comparisons.

### NMR Dataset

To assess the conformational variability captured in the dataset, we analyzed the maximum per-atom standard deviation of chemical shifts across molecular dynamics frames, computed separately for each molecule. For every molecule, we identified the atom (of type H or C) with the highest standard deviation in its chemical shift, reflecting the most dynamically responsive local environment.

**Fig. 9:**
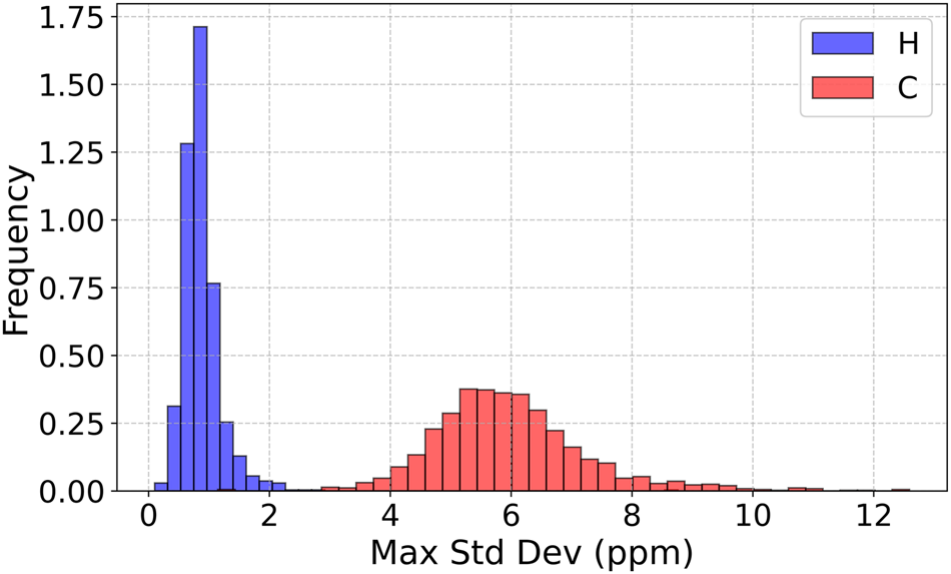
Distribution of the maximum standard deviation of chemical shifts per atom across molecular dynamics frames for each molecule. Two histograms show the frequency for hydrogen (H, blue) and carbon (C, red) atoms. Each value represents the largest variation in NMR chemical shift among all atoms of a given element within a single molecule, reflecting local flexibility or sensitivity to conformational changes.

Figure 9 shows the resulting distribution for hydrogen (blue) and carbon (red) atoms. For hydrogen atoms, most molecules exhibit a maximum standard deviation below 2 ppm, with a sharp peak near 1 ppm. In contrast, carbon atoms show a significantly broader and higher-variance distribution, with peaks around 5-6 ppm and tails extending beyond 10 ppm. The maximum per-atom standard deviation across MD frames varies significantly, especially for carbon atoms. This highlights the necessity of averaging over conformations: relying on a single structure can yield noisy or non-representative chemical shift values due to transient molecular fluctuations.

This approach also mirrors experimental reality: in solution-state NMR spectroscopy, chemical shifts reflect an ensemble average over rapidly interconverting conformations. Thus, the use of time-averaged values in our dataset is both practically and physically justified, ensuring greater fidelity to experimental observables and enhancing the robustness of downstream learning tasks.

**Fig. 10:**
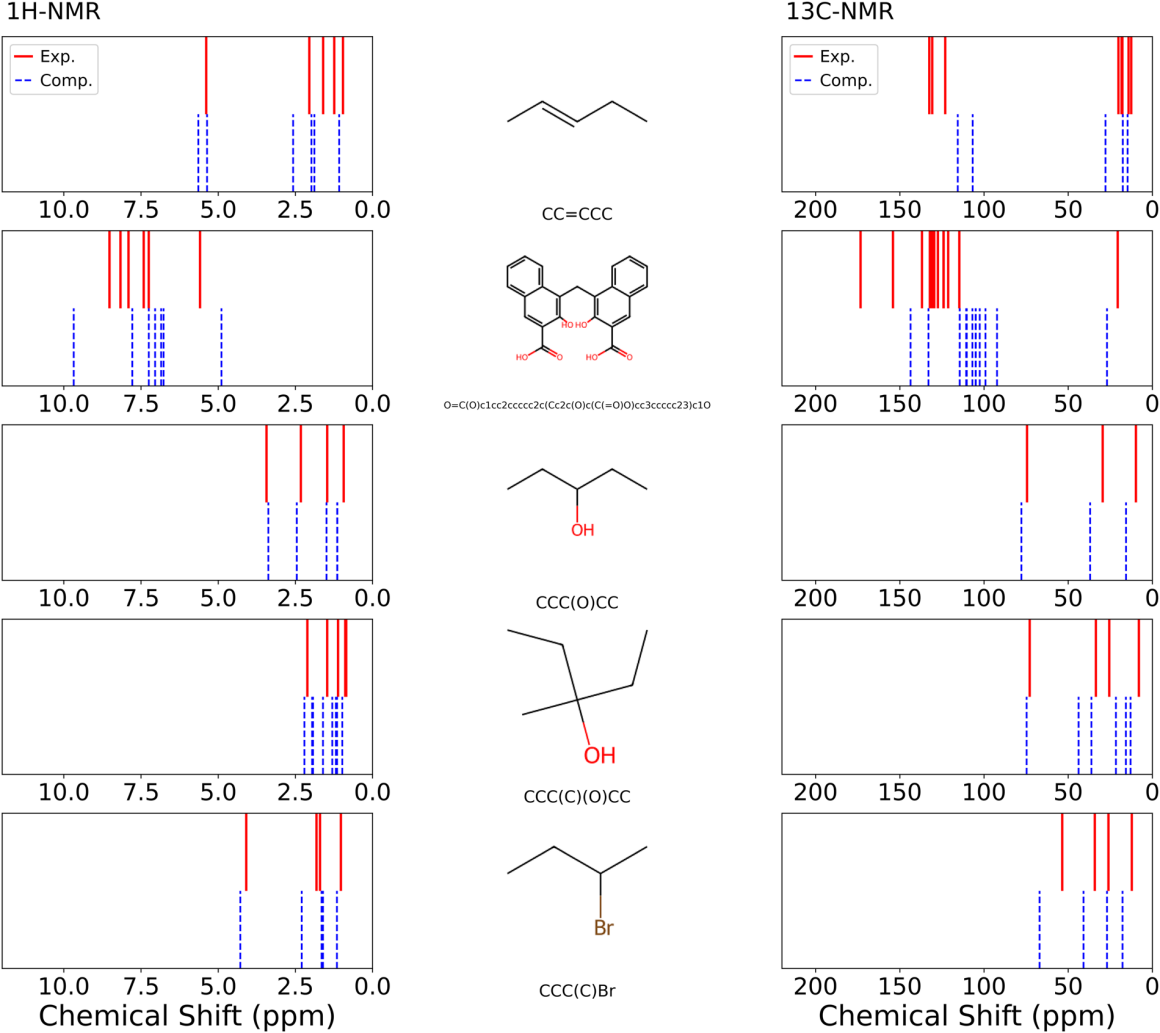
Comparison of experimental and computed NMR spectra for five representative molecules from the dataset. For each molecule, the molecular structure (center) is flanked by the ^1^H-NMR (left) and ^13^C-NMR (right) spectra are shown. Red lines indicate experimental (Exp.) chemical shifts, while blue dashed lines correspond to computed (Comp.) chemical shifts obtained from molecular dynamics sampling. All spectra share a consistent chemical shift axis to allow direct comparison across molecules.

Figure 10 provide a comparison for five representative molecules, displaying their molecular structures alongside experimental data from the “Spectroscopy Database”^8^ described in Ref. ^36^ and computed ^1^H-NMR and ^13^C-NMR spectra to illustrate typical agreement and deviations. Figures S2 and S3 in the Supporting Information show a comparison of experimental and computed ^1^H-NMR and ^13^C-NMR chemical shifts for a representative set of 15 additional molecules.

**Fig. 11:**
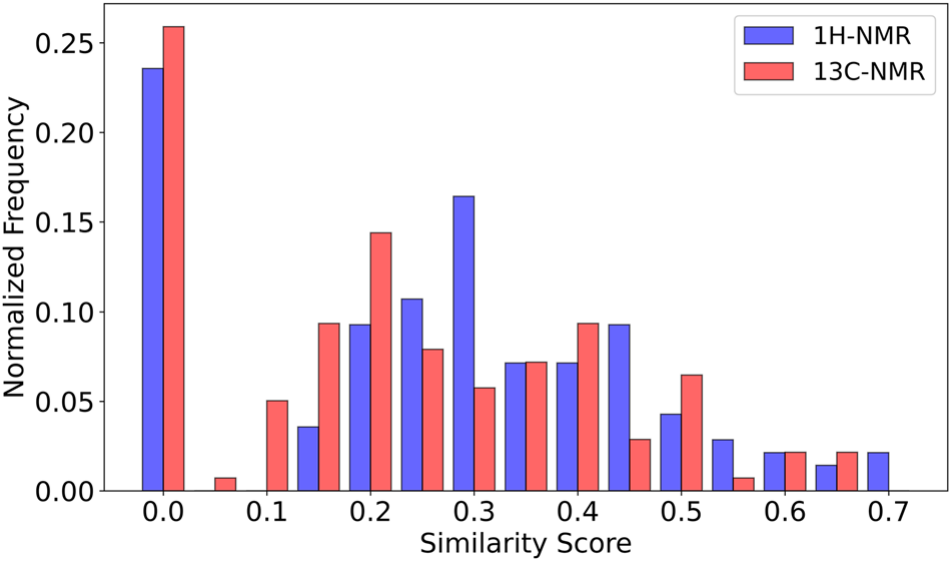
Normalized frequency of similarity scores between DFT-computed^16^ and experimental NMR spectra^8^ for 140 overlapping molecules present in both datasets. Similarity scores were calculated using cosine similarity with tolerances of 0.1 ppm for ^1^H-NMR (blue bars) and 2.0 ppm for ^13^C-NMR (red bars). Higher scores indicate better agreement between computed and experimental chemical shifts, with scores approaching 1.0 representing near-perfect matches.

To quantify the overall agreement between computed and experimental NMR data across the 140 common molecules of the experimental dataset^8^ of Ref. ^36^ and the computed NMR data from our dataset^16^, we calculated similarity scores using cosine similarity as in Ref. ^17^ with chemical shift tolerances of 0.1 ppm for ^1^H-NMR and 2.0 ppm for ^13^C-NMR, reflecting the approximately 20-fold difference in typical chemical shift ranges between the two nuclei, see Fig. 10. Figure 11 shows the distribution of these similarity scores, providing a statistical overview of computational accuracy beyond the qualitative comparisons presented above.

## Usage Notes

This dataset serves multiple purposes across computational spectroscopy and machine learning applications.

### Infrared (IR) Dataset for Spectroscopy

The IR dataset can be used to benchmark anharmonic spectral calculations against traditional harmonic approximation methods and to evaluate the performance of molecular dynamics (MD)-based approaches in capturing vibrational anharmonicity. It also supports the training and fine-tuning of machine learning models aimed at predicting IR spectra beyond the harmonic approximation. Additionally, the dataset provides high-quality theoretical references that can assist experimentalists in interpreting complex IR spectra.

### Nuclear Magnetic Resonance (NMR) Spectroscopy

For NMR applications, the dataset enables benchmarking of DFT methods for predicting hydrogen (^1^H-NMR) and carbon (^13^C-NMR) chemical shifts. It is also well-suited for training or fine-tuning machine learning models that aim to deliver fast and accurate chemical shift predictions. Furthermore, it facilitates molecular structure assignment by allowing direct comparison between computed and experimental NMR spectra.

### General Applications

Beyond specific spectroscopic domains, the dataset can contribute to the development of hybrid ML-DFT methods that improve spectral accuracy while lowering computational cost. It supports advances in automated molecular characterization by enabling the integration of IR and NMR spectral information. Finally, it provides a high-quality reference resource for testing and benchmarking emerging methodologies in computational spectroscopy.

This dataset is structured for flexible usage—whether for standalone IR or NMR analysis or for combined studies aimed at gaining deeper insight into molecular structure and dynamics.

## Supplementary information

Supporting Information of IR-NMR multimodal computational spectra dataset for 177K patent-extracted organic molecules

## Data Availability

Code used for extracting and analyzing the data is available at GitHub: https://github.com/rxn4chemistry/MultimodalAnalytical, particularly under paper_replication/scripts_ir_nmr_multimodal_comp_spectra_dataset/. Please refer to the README.md at https://github.com/rxn4chemistry/MultimodalAnalytical/tree/main/paper_replication/scripts_ir_nmr_multimodal_comp_spectra_dataset for setup instructions and replication details. The code is available under the MIT license.

## References

[1] Lu, X.-Y. *et al*. Deep Learning-Assisted Spectrum-Structure Correlation: State-of-the-Art and Perspectives. *Analytical Chemistry* **96**, 7959–7975 (2024). 38662943 10.1021/acs.analchem.4c01639

[2] Linstrom, P. J. & Mallard, W. G. The NIST Chemistry WebBook: A Chemical Data Resource on the Internet. *Journal of Chemical and Engineering Data* **46**, 1059–1063 (2001).

[3] National Institute of Standards and Technology (NIST) NIST Chemistry WebBook Standard Reference Database 69. Specifically, IR spectra from the Coblentz Society collection. Accessed: July 21, 2025. https://webbook.nist.gov/chemistry/ (2001).

[4] Chu, P. M., Guenther, F. R., Rhoderick, G. C. & Lafferty, W. J. The NIST Quantitative Infrared Database. *Journal of Research of the National Institute of Standards and Technology* **104**, 59 (1999). NIST Standard Reference Database 79 (SRD 79). This article serves as the primary citation for the dataset. Accessed: July 21 (2015).

[5] National Institute of Advanced Industrial Science and Technology (AIST) SDBS Spectral Database for Organic Compounds. n.d.; Accessed: July 21, https://sdbs.db.aist.go.jp/ (2015).

[6] John Wiley & Sons, Inc. KnowItAll Spectroscopy Edition and Spectral Libraries. n.d.; Specifically, IR spectral collections. Accessed: July 21, https://sciencesolutions.wiley.com/solutions/technique/ir/knowitall-ir-collection/ (2015).

[7] Kuhn, S. & Schlörer, N. E. Facilitating quality control for spectra assignments of small organic molecules: nmrshiftdb2 – a free in-house NMR database with integrated LIMS for academic service laboratories. *Magnetic Resonance in Chemistry* **53**, 582–589 (2015). 25998807 10.1002/mrc.4263

[8] Van Bramer, S. E. & Bastin, L. D. Spectroscopy Database. 10.6084/m9.figshare.c.6754047.v1 (2023).

[9] Zhang, R. *et al*. 2DNMRGym: An Annotated Experimental Dataset for Atom-Level Molecular Representation Learning in 2D NMR via Surrogate Supervision. arXiv preprint arXiv:2505.18181, http://arxiv.org/abs/2505.18181 (2025).

[10] Verma, S. *et al*. 2DNMRGym Dataset. https://github.com/siriusxiao62/2DNMRGym (2025).

[11] Bagheri, M. & Komsa, H.-P. High-throughput computation of Raman spectra from first principles. *Scientific Data* **10**, 80 (2023). 36755025 10.1038/s41597-023-01988-5 PMC9908888

[12] Li, Y. *et al*. A database of computed Raman spectra of inorganic compounds with accurate hybrid functionals. *Scientific Data* **11**, 105 (2024). 38253529 10.1038/s41597-024-02924-x PMC10803741

[13] Li, G. *et al*. High-Throughput Computation of ab initio Raman Spectra for Two-Dimensional Materials. *Scientific Data* **12**, 373 (2025). 40038321 10.1038/s41597-025-04593-w PMC11880192

[14] Liang, Q., Dwaraknath, S. & Persson, K. A. High-throughput computation and evaluation of Raman spectra. *Scientific Data* **6**, 135 (2019). 31350415 10.1038/s41597-019-0138-y PMC6659686

[15] Liang, J., Ling, J., Xu, L. & Zhu, X. A Dataset of Raman and Infrared Spectra as an Extension to the ChEMBL. *Scientific Data* **12**, 939 (2025). 40467651 10.1038/s41597-025-05289-x PMC12137555

[16] Zipoli, F.; Alberts, M.; Laino, T. IR-NMR Multimodal Computational Spectra Dataset for 177K Patent-Extracted Organic Molecules. 10.5281/zenodo.15669241 (2025). 10.1038/s41597-025-05729-8 PMC12331906 40775416

[17] Alberts, M.; Schilter, O.; Zipoli, F.; Hartrampf, N.; Laino, T. Unraveling molecular structure: A multimodal spectroscopic dataset for chemistry. Advances in Neural Information Processing Systems. 125780–125808 (2024).

[18] Lowe, D. Chemical reactions from US patents (1976-Sep2016) (2017).

[19] Lowe, D. M. Extraction of chemical structures and reactions from the literature. Ph.D. thesis, University of Cambridge (2012).

[20] Landrum, G.; others RDKit: Open-source cheminformatics. Accessed: 2025-04-06, https://www.rdkit.org (2024).

[21] He, X., Man, V. H., Yang, W., Lee, T.-S. & Wang, J. A fast and high-quality charge model for the next generation general AMBER force field. *The Journal of Chemical Physics* **153**, 114502 (2020). 32962378 10.1063/5.0019056 PMC7728379

[22] Thompson, A. P. *et al*. LAMMPS - a flexible simulation tool for particle-based materials modeling at the atomic, meso, and continuum scales. *Comp. Phys. Comm.* **271**, 108171 (2022).

[23] Car, R. & Parrinello, M. Unified Approach for Molecular Dynamics and Density-Functional Theory. *Physical Review Letters* **55**, 2471–2474 (1985). 10032153 10.1103/PhysRevLett.55.2471

[24] CPMD Developers CPMD, a parallelized plane wave / pseudopotential implementation of DFT. MPI für Festkörperforschung Stuttgart and IBM Zurich Research Laboratory, https://www.github.com/CPMD-code (1990).

[25] Perdew, J. P., Burke, K. & Ernzerhof, M. Generalized Gradient Approximation Made Simple. *Physical Review Letters* **77**, 3865–3868 (1996). 10062328 10.1103/PhysRevLett.77.3865

[26] Goedecker, S., Teter, M. & Hutter, J. Separable dual-space Gaussian pseudopotentials. *Phys. Rev. B* **54**, 1703–1710 (1996). 10.1103/physrevb.54.1703 9986014

[27] Marzari, N. & Vanderbilt, D. Maximally localized generalized Wannier functions for composite energy bands. *Phys. Rev. B* **56**, 12847–12865 (1997).

[28] Wang, H., Zhang, L. & Han, J. DeePMD-kit: A deep learning package for many-body potential energy representation and molecular dynamics. *Computer Physics Communications* **228**, 178–184 (2018).

[29] Zeng, J. *et al*. DeePMD-kit v2: A software package for Deep Potential models. *The Journal of Chemical Physics* **159**, 054801 (2023). 37526163 10.1063/5.0155600 PMC10445636

[30] Zhang, L. *et al*. Deep neural network for the dielectric response of insulators. *Phys. Rev. B* **102**, 041121 (2020).

[31] Lu, D. *et al*. 86 PFLOPS Deep Potential Molecular Dynamics simulation of 100 million atoms with ab initio accuracy. *Comput. Phys. Comm.* **259**, 107624 (2021).

[32] Zhang, L. *et al*. End-to-end Symmetry Preserving Inter-atomic Potential Energy Model for Finite and Extended Systems. *Advances in Neural Information Processing Systems* **31**, 4436–4446 (2018).

[33] Braun, E. *Calculating an IR Spectra from a LAMMPS Simulation*. 10.5281/zenodo.154672 (2016).

[34] Blackman, R.; Tukey, J. *The Measurement of Power Spectra from the Point of View of Communications Engineering*; Dover Publications (1958)

[35] Gaigeot, M.-P. & Sprik, M. Ab Initio Molecular Dynamics Computation of the Infrared Spectrum of Aqueous Uracil. *The Journal of Physical Chemistry B* **107**, 10344–10358 (2003).

[36] Van Bramer, S. E. & Bastin, L. D. Spectroscopy Data for Undergraduate Teaching. *Journal of Chemical Education* **100**, 3897–3902 (2023).

