# Supplementary material for: IR-NMR multimodal computational spectra dataset for 177K patent-extracted organic molecules
Source: Sci Data. 2025 Aug 7;12:1375. doi: 10.1038/s41597-025-05729-8 (PMC12331906; doi:10.1038/s41597-025-05729-8)
Supplement: Supplementary file 1 — Supporting Information of IR-NMR multimodal computational spectra dataset for 177K patent-extracted organic molecules [file 41597_2025_5729_MOESM1_ESM.pdf]

Supporting Information of IR-NMR multimodal computational spectra dataset for 177K patent-extracted organic molecules

Federico Zipoli,[*,†,‡] Marvin Alberts,[†,‡] and Teodoro Laino[†,‡]

†*IBM Research Europe, Saümerstrasse 4, 8803 Rüschlikon, Switzerland*

‡*NCCR Catalysis, Switzerland*

E-mail: fzi@zurich.ibm.com

UMAP projection of Morgan fingerprints for the scaffold molecules

Figure S1 shows a UMAP projection of Morgan fingerprints for the scaffold molecules. In the right panel, the subset of molecules used for dipole training is overlaid in red to illustrate coverage in the embedding space.

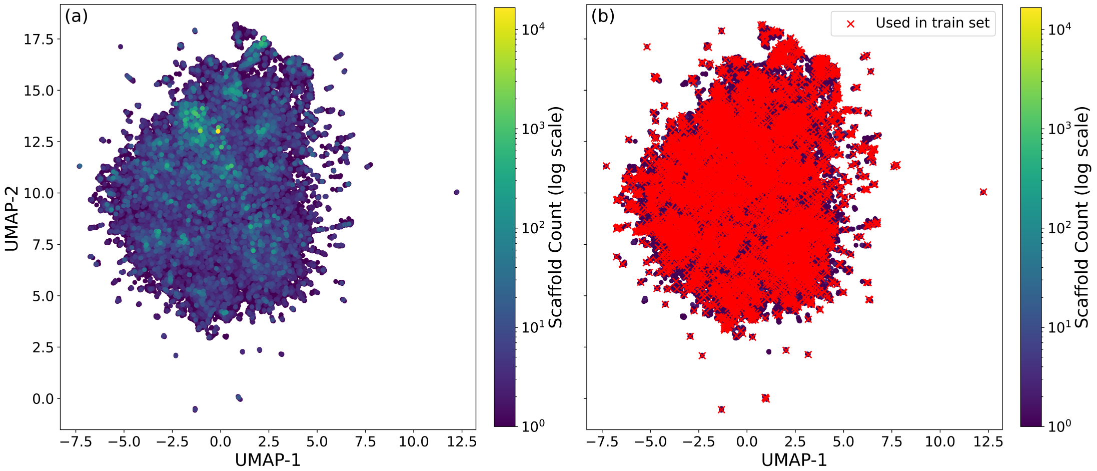

Figure S1: UMAP visualization of Morgan fingerprints for scaffold molecules. (a) The left panel shows all molecules in the dataset. (b) The right panel overlays in red the subset used for training the dipole moment model.

LAMMPS Input Script Used for Dipole Calculations

Listing 1: LAMMPS input for Langevin equilibration and dipole moment extraction

```
commentstyle
# ----------------- Init -----------------
units real
atom_style full
neigh_modify delay 0 every 1
pair_style lj/cut/coul/long 12.0
bond_style harmonic
angle_style harmonic
dihedral_style harmonic
special_bonds amber
kspace_style pppm 1e-5
read_data data.lammps
# ----------------- Equilibration -----------------
timestep 0.5
thermo 200
thermo_style custom step temp ke pe etotal time tpcpu cpu
write_dump all custom initial_state.xyz id type x y z
run 0
velocity all create 300 38564
fix temp all langevin 300 300 100 1530917
fix 1 all nve
fix 2 all recenter 25. 25. 25.
fix 3 all momentum 1000 linear 1 1 1 angular
run 50000

write_dump all custom final_end_langevin_state.xyz id type x y z vx vy vz
unfix temp
unfix 2
unfix 3
reset_timestep 0

# ----------------- Production -----------------
compute 1 all property/atom q xu yu zu
variable dipolex atom c_1[1]*c_1[2]
variable dipoley atom c_1[1]*c_1[3]
variable dipolez atom c_1[1]*c_1[4]
compute 2 all reduce sum v_dipolex
compute 3 all reduce sum v_dipoley
compute 4 all reduce sum v_dipolez
variable totaldipolex equal c_2
variable totaldipoley equal c_3
variable totaldipolez equal c_4
variable mytime equal step*dt
fix printdipole all ave/time 1 1 2 v_mytime v_totaldipolex v_totaldipoley v_totaldipolez
    file dipole.txt mode scalar

thermo_style custom step temp ke pe etotal time
thermo 200
dump traj2 all xyz 5 pos_every_2p5fs.xyz
run 200000 # 100 ps (timestep is 0.5 fs)
```

DeePMD-kit Input Script Used to Train Dipole Predictions

Listing 2: List of parameters used to train the model for dipole prediction.

```
commentstyle
{
  "_comment1": " model parameters",
  "model": {
    "type_map": [ "B", "Br", "C", "Cl", "F", "H", "I", "N", "O", "P", "S", "Si" ],
    "descriptor": {
      "type": "se_e2_a",
      "sel": [ 66, 60, 84, 68, 62, 84, 66, 78, 84, 84, 68, 64 ],
      "rcut_smth": 1.00,
      "rcut": 6.00,
      "neuron": [ 32, 64, 128 ],
      "resnet_dt": true,
      "axis_neuron": 16,
      "type_one_side": true,
      "precision": "float32",
      "seed": 1
    },
    "fitting_net": {
      "type": "dipole",
      "sel_type": [ 0, 1, 2, 3, 4, 5, 6, 7, 8, 9, 10, 11 ],
      "neuron": [
        128,
        128,
        128
      ],
      "resnet_dt": true,
      "precision": "float32",
      "seed": 1
    }
  },
  "learning_rate": {
    "type": "exp",
    "start_lr": 1.0e-4,
    "decay_steps": 5000000
  },
  "loss": {
    "type": "tensor",
    "pref": 1.0,
    "pref_atomic": 1.0
  },
  "training": {
    "training_data": {
      "systems": [
        "../data/100006/training_data_reformat/global_system",
        "../data/100006/training_data_reformat/atomic_system",
         [...]
      ],
      "batch_size": 20
    },
```

```
    "validation_data": {
      "systems": [
        "../data/104680/validation_data_reformat/global_system",
        "../data/104680/validation_data_reformat/atomic_system",
         [...]
      ],
      "batch_size": 3,
      "numb_btch": 3
    },
    "numb_steps": 5000000,
    "seed": 10,
    "disp_file": "lcurve.out",
    "disp_freq": 100,
    "save_freq": 10000,
    "max_ckpt_keep": 100
  }
}
```

Validation of Computed NMR Chemical Shifts

This section presents a detailed comparison between experimentally measured and computationally predicted nuclear magnetic resonance (NMR) chemical shifts for a representative set of 15 organic molecules. Proton (^{1}H) and Carbon-13 (^{13}C) NMR spectra were simulated by averaging chemical shifts obtained from molecular dynamics sampling, providing a dynamic and realistic representation of the molecular environments. The figures S2 and S3 show the correlation between experimental data (red vertical lines) and computed averaged peaks (blue dashed lines) for each molecule, identified by their SMILES notation. The x-axes are displayed in the conventional decreasing ppm scale, facilitating direct comparison with experimental NMR spectra.

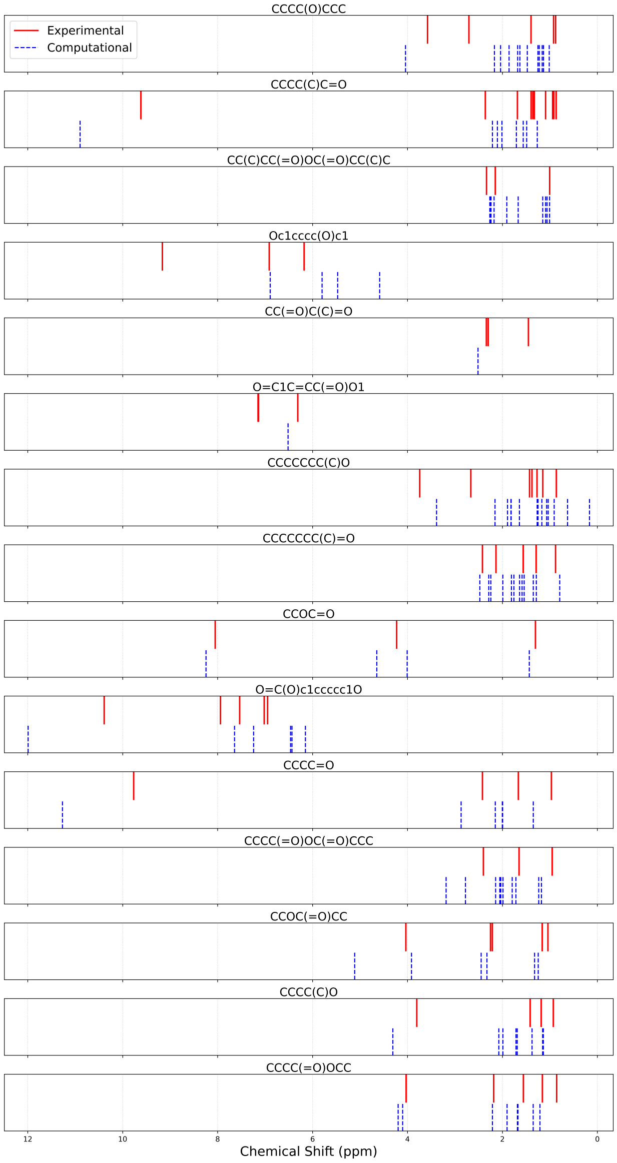

Figure S2: Comparison of experimental and computed H-NMR peaks for 15 molecules. Vertical red lines indicate experimental chemical shifts, and blue dashed lines show computed averaged H-NMR peaks from molecular dynamics sampling. Each subplot corresponds to a distinct molecule, labeled with its SMILES string. The x-axis is displayed in decreasing ppm, consistent with NMR conventions.

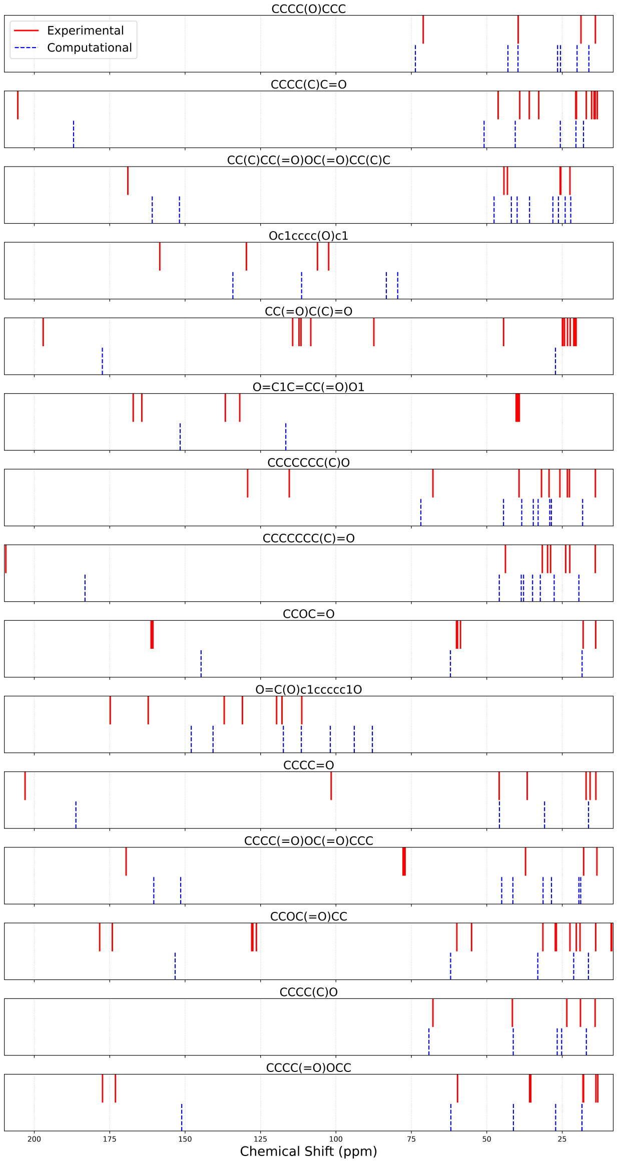

Figure S3: Comparison of experimental and computed ^{13}C-NMR peaks for 15 molecules. Vertical red lines indicate experimental chemical shifts, and blue dashed lines show computed averaged ^{13}C-NMR peaks from molecular dynamics sampling. Each subplot corresponds to a distinct molecule, labeled with its SMILES string. The x-axis is displayed in decreasing ppm, consistent with NMR conventions.

NMR Shielding Tensors from CPMD Calculations

As part of this study, we computed ^{13}C and ^{1}H nuclear magnetic shielding tensors using CPMD code. The shielding constants are reported for a representative molecule (SMILES: `CC=CCC`), and presented below in Table S1 for each atom in the structure. The values correspond to the principal components of the shielding tensor (PV1, PV2, PV3), their isotropic average (iso), the anisotropy (aniso), and the reference-shifted isotropic shielding (TMS_ref-iso), relative to a computed TMS shielding.

These data are stored as plain text output by the CPMD code and are programmatically accessible via the tag `nmr_cpmd_text`. We reference this in the main text as a typical example for interpretation of calculated NMR parameters.

Table S1: NMR shielding tensor components computed with CPMD for `CC=CCC`.

Atom	Index	PV1	PV2	PV3	iso	aniso	TMS_ref-iso
C	1	-11.40	-4.69	12.08	-1.34	20.12	13.07
C	2	-155.65	-110.33	-11.09	-92.36	121.91	104.09
C	3	-182.93	-89.53	-31.45	-101.30	104.78	113.03
C	4	-31.84	-16.78	-7.91	-18.84	16.40	30.57
C	5	-19.58	-12.30	8.61	-7.76	24.55	19.49
H	1	23.67	28.98	32.78	28.48	6.45	1.91
H	2	25.38	26.77	35.17	29.11	9.10	1.28
H	3	24.44	27.53	35.15	29.04	9.16	1.35
H	4	20.88	26.23	27.67	24.93	4.12	5.46
H	5	22.87	24.07	26.11	24.35	2.65	6.04
H	6	23.81	27.20	33.96	28.32	8.46	2.07
H	7	23.12	27.49	31.22	27.28	5.91	3.11
H	8	26.27	27.81	35.80	29.96	8.76	0.43
H	9	23.54	25.92	34.31	27.92	9.58	2.47
H	10	25.59	27.56	34.75	29.30	8.17	1.09
